# ID 223 - Método Denver para Transtorno do Espectro Autista: revisão sistemática de ensaios clínicos randomizados

**DOI:** 10.5327/2237-9622.2025.v34s1.223

**Published:** 2025-11-25

**Authors:** Camila Monteiro Cruz, Camila Monteiro Cruz, Ana Luiza Cabrera Martimbianco, Carolina de Oliveira Cruz Latorraca, Cecília Menezes Farinasso, Isabela Porto de Toledo, Patrícia do Carmo Silva Parreira, Rafael Leite Pacheco, Roberta Borges Silva, Verônica Colpani, Rachel Riera

**Keywords:** transtorno do espectro autista, TEA, Denver, eSDM, revisão sistemática

## Abstract

**Introdução:**

A prevalência de transtorno do espectro autista (TEA) no Brasil e no mundo é estimada em 1%, e dados do Sistema Único de Saúde (SUS) apontam que essas pessoas realizaram 9,6 milhões de atendimentos ambulatoriais em 2021. O método Denver é uma terapia comportamental naturalista, individualizada, aplicada em ambientes não simulados (como escola e domicílio, por exemplo), que utiliza os princípios da teoria da aprendizagem, como o reforço positivo, com foco no estímulo de comportamentos desejados e no controle dos comportamentos indesejados. O objetivo deste estudo foi avaliar os efeitos do método Denver para pessoas com TEA.

**Método:**

Revisão sistemática, conduzida no Núcleo de Avaliação de Tecnologias em Saúde e Núcleo de Evidências do Hospital Sírio-Libanês (Nats/NEv-HSL), seguindo as recomendações metodológicas do e relatada de acordo com o (PRISMA 2020). O protocolo foi registrado prospectivamente na base PROSPERO (https://www.crd.york.ac.uk/prospero/display_record.php?RecordID=534723). Em 13 de abril de 2024, foi realizada busca ampla e sensível em bases eletrônicas (ADOLEC, CENTRAL, Embase, LILACS, MEDLINE, PsycNET), literatura cinzenta (DANS), registros de ensaios clínicos (clinicaltrials.gov e ICTRP-WHO) e busca manual em listas de referências. Foram considerados ensaios clínicos randomizados (ECR) que avaliaram o método Denver (original ou adaptado) para pessoas com TEA comparado com nenhuma intervenção, , lista de espera, outras psicoterapias ou terapias múltiplas. Desfechos primários: melhora global e gravidade dos sintomas; secundários: interação social, comportamento adaptativo e social, comunicação verbal e não verbal, qualidade de vida, habilidade cognitiva, estereotipia, satisfação e avaliação dos cuidadores e eventos adversos. O risco de viés dos ECR foi avaliado com a tabela de risco de viés da Cochrane, e a certeza da evidência foi avaliada pela abordagem GRADE (do inglês,).

**Resultados:**

Foram identificadas 6.038 referências e, após o processo de seleção, 13 ECR foram incluídos (9 com resultados disponíveis e totalizando 722 participantes). Os desfechos avaliados pelos ECR foram: avaliação global, gravidade dos sintomas, interação social, comportamento adaptativo e social, comunicação verbal e não verbal e habilidade cognitiva. Considerando todas as comparações, a certeza da evidência para todos os desfechos foi "muito baixa", indicando que há incerteza quanto aos benefícios e riscos do método ABA para TEA. A certeza nas evidências foi reduzida pelo alto risco de viés dos ECR incluídos e pela imprecisão dos resultados.

Sínteses quantitativas não foram possíveis devido à heterogeneidade clínica dos ECR, considerando a diversidade das modalidades de Denver utilizadas, dos desfechos avaliados, das ferramentas utilizadas para mensuração e o relato insuficiente dos resultados numéricos.

**Conclusão:**

De acordo com os resultados dos ECR disponíveis até o momento, os benefícios e riscos do método Denver para o tratamento de pessoas com TEA, quando comparado a nenhum tratamento, lista de espera, outras psicoterapias ou terapias múltiplas, são incertos. Diante dessa incerteza, a tomada de decisão deve considerar a existência de alternativas terapêuticas disponíveis para o caso, a disponibilidade de profissionais capacitados e devidamente treinados no método Denver, a heterogeneidade da aplicação do método e o desconhecimento sobre os efeitos e riscos do método também no longo prazo.

